# Intestinal Parasitic Infections in Ghana: A Review of Infections and the Risk of Zoonotic Transmission

**DOI:** 10.1155/bmri/7766777

**Published:** 2025-09-01

**Authors:** Seth Offei Addo, Margaret Owusu-Akyaw, Ronald Essah Bentil, Stacy Amoah, Seth Oware, Bernice Olivia Ama Baako, John Asiedu Larbi

**Affiliations:** ^1^ Parasitology Department, Noguchi Memorial Institute for Medical Research, University of Ghana, Accra, Ghana, ug.edu.gh; ^2^ Department of Theoretical and Applied Biology, Kwame Nkrumah University of Science and Technology, Kumasi, Ghana, knust.edu.gh; ^3^ Department of Medical Microbiology, University of Ghana Medical School, University of Ghana, Accra, Ghana, ug.edu.gh; ^4^ Department of Biological Science, University for Development Studies, Tamale, Ghana, uds.edu.gh; ^5^ Navrongo Health Research Centre, Navrongo, Upper East Region, Ghana, navrongo-hrc.org

## Abstract

Intestinal parasites pose a risk to public health globally, causing a high economic burden in developing countries. Most diagnostic methods aimed at detecting these parasites in hospital settings are not sensitive to recovering parasites. Hence, the prevalence of these diseases remains a challenge in areas of poor environmental sanitation and inadequate diagnostic tools. Control strategies are critical but may need a deep understanding of the epidemiology of parasitic diseases. In Ghana, intestinal parasitic infections are considered to be an important public health issue. Despite efforts to reduce the burden of infections, these parasites continue to spread. As such, this review is aimed at bringing up to date the existing information on the burden of intestinal parasites in Ghana by compiling available publications from 1975 to 2023. Emphasis is made on parasitic infections in vulnerable groups, livestock and the risk of zoonotic transmission. There is a need for more surveillance efforts to determine the dynamics of infections as well as intensify mass education.

## 1. Background

Intestinal parasitic infections constitute a considerable public health issue with about 3.5 billion people being infected globally [1]. The majority of infections have been reported in developing countries as a result of poor sanitation conditions, sparse water supply and rapid population growth [2, 3]. Annual reports estimate about 10.5 million new cases with *Ascaris lumbricoides*, hookworms, *Trichuris trichiura*, *Giardia duodenalis*, *Entamoeba histolytica* and *Schistosoma* species being implicated as the predominant intestinal parasites [4]. In Africa, available estimates suggest that over 198 million people are likely to be infected with hookworm, 173 million with *A. lumbricoides* and 162 million with *T. trichiura* [5]. In countries with the prevalence of diarrhoea, *G. duodenalis* is the culprit parasitic agent responsible for infections [6]. According to the World Health Organization (WHO), amoebiasis, a disease caused by the parasite *E. histolytica*, is responsible for thousands of deaths yearly, with about 50 million people suffering from invasive amoebic infection [7]. Cryptosporidiosis has become endemic in both developed and developing countries; people with AIDS and children below the age of 5 years are believed to be most susceptible [8, 9]. Within the last decade, there have been reports of several diarrhoea outbreaks owing to increased *Cyclospora cayetanensis* infections [10]. Intestinal parasites are transmitted through the consumption of contaminated food and having contact with infected soil [11]. Transmission can also occur through active skin penetration by the larvae of the parasite [12]. Thus, farmers, who are often in contact with soil, as well as children and pregnant women, are at a higher risk of being infected [13]. Due to their behaviours of eating with dirty hands, playing in contaminated soils, using unsanitary toilets and ingesting contaminated food and drink orally, children under the age of 5 bear the greatest burden of intestinal parasites [14].

Intestinal parasitic infections are a recognized public health issue in Ghana [15–17]. The Ministry of Health responds to this burden with a dynamic policy that includes a school‐age deworming programme that is ongoing [18]. The results of Ghana’s periodic deworming programme may be satisfactory, as evidenced by the relatively low incidence of infections found in certain investigations [15, 19–21]. However, it is crucial to continually investigate the occurrence of intestinal parasites amongst patients who present to the hospital and the communities at large to accurately determine the burden of infections and the effects of periodic deworming.

### 1.1. Diagnosis of Intestinal Parasites

In the fight against intestinal protozoan parasites, investigators have formulated new diagnostic methods. The use of the traditional method, the direct smear method, in hospital settings although quick, economical and able to identify parasites in a watery stool sample is not reliable as most parasites are not recovered. In light of this failure, it is necessary to employ other diagnostic methods for intestinal parasite detection, and this includes the use of antigen detection tests, Kato–Katz method and formol‐ether concentration (FEC) as confirmatory tests after diagnosis with the direct smear method. The Kato–Katz method is effective in the survey of intestinal helminths and *Schistosoma mansoni* [22]. The FEC method is useful in the recovery of ova/egg, cyst and larvae and conserves the morphology of intestinal parasites for easy identification, especially in concentrated stool samples [23]. Antigen detection methods were created specifically for detecting protozoan parasites such as *E. histolytica* [24, 25], *G. duodenalis* [26] and *Cryptosporidium* spp. [27, 28]. In addition, there have been reports of enzyme‐linked immunosorbent assays (ELISA), modified Ziehl–Neelsen technique and multiparallel and multiplex PCR‐based diagnostic methods for the detection of intestinal parasites [29, 30]. This approach enhanced the detection of multiple parasite species from a single stool sample as well as determining the intensity of infection of each species [31, 32] but comes at a relatively higher cost. In Ghana, hospitals depend mostly on the microscopic analysis of the faeces to identify intestinal parasites. In the face of limited resources, most infections caused by intestinal parasites will go undetected and unreported. There is a need to develop more effective diagnostic methods in the various hospital facilities across Ghana to ensure the timely detection of intestinal parasitic infections which will lead to more effective treatment outcomes.

### 1.2. Methods of Search

In this literature review, published data on intestinal parasites in Ghana from 1975 to 2024 were searched from PubMed, Google Scholar and Scopus. The search for studies was conducted using phrases such as intestinal parasites in Ghana, intestinal parasites in children, intestinal parasites in pregnant women, intestinal parasites in vulnerable groups, intestinal parasitic infections in animals and the risk of zoonotic transmission and intestinal parasites in fruits and vegetables.

## 2. Intestinal Parasitic Infections in Ghana

Studies conducted over the years indicate the continuous spread of intestinal parasitic infections within the country [33–36]. Although there have been deworming exercises in Ghana, its coverage has been limited and irregular; hence, the risk of infection remains a threat to the entire population [16]. The risk groups in Ghana include vulnerable populations such as children, prisoners, pregnant women and individuals with compromised immune systems [37]. The reports indicate the persistence of intestinal parasites in the country and a need to adopt effective control and preventive measures (Table 1).

**Table 1:** Intestinal parasitic infections in Ghana.

**Location**	**Diagnostic method**	**Sample source**	**Helminths/protozoa**	**Overall prevalence**	**References**
Kintampo North Municipality and Kintampo South District	Direct wet mount, Kato–Katz, formol‐ether‐based concentration	Humans (above 6 months)	Helminths	19.30%	Adu‐gyasi et al. [38]
Ga West Municipality	Direct wet mount, formol‐ether concentration	Humans (6 years and above)	Helminths and protozoa	33.10%	Aninagyei et al. [39]
Sekyere	Formol‐ether concentration	Humans (pregnant women aged 12–45)	Helminths	17.60%	Baidoo et al. [33]
Kintampo North Municipality	Kato–Katz	Humans (1–80 years)	Helminths	45%	Humphries et al. [34]
Kintampo North Municipality	Kato–Katz	Humans (school children 6–11 years)	Helminths	39.10%	Humphries et al. [35]
Accra	Direct wet mount	Humans (children 0–10 years)	Helminths	17.30%	Mirisho et al. [16]
Bawku District	Formol‐ether concentration, Kato–Katz, PCR	Humans	Helminths and protozoa	39.80%	Verweij et al. [40]
Berekum	Direct wet mount	Humans (women)	Helminths and protozoa	21.20%	Deku et al. [41]
Southern Ghana	Formol‐ether concentration and Ziehl–Neelsen technique	Humans (prison inmates)	Helminths and protozoa	38.20%	Abaka‐Yawson et al. [42]
Rural villages in Ghana	Telemann method	Humans (preschool children)	Helminths and protozoa	—	Annan et al. [43]
Accra	Direct wet mount, modified Ziehl–Neelsen	Humans (children > 5 years)	Protozoa	27.8% and 15.6% in children with and without diarrhoea, respectively	Adjei et al. [44]
Accra	Direct wet mount, ELISA, PCR	Humans (children ≤ 5 years)	Protozoa	5.8% and 5% in diarrhoea and nondiarrhoea cases, respectively	Anim‐Baidoo et al. [45]
Awutu‐Efutu‐Senya District	Kato–Katz	Humans (infants 1–5 years)	Helminth	4%	Bosompem et al. [46]
Accra	Direct wet mount	Humans (school children 2–9 years)	Helminths and protozoa	15%	Forson et al. [15]
Cape Coast metropolis	Direct wet mount	Humans (school children 6–17 years)	Helminths and protozoa	19.10%	Dankwa et al. [47]
Kassena‐Nankana East and West Districts	Direct wet mount and formol‐ether concentration	Humans (school children 5–15 years)	Helminth	2.79% for direct wet mount and 9.40% for formol‐ether concentration	Sam et al. [48]
Volta region	Direct wet mount	Humans (school children 6–14 years)	Helminth	2.36%	Orish et al. [21]
Ho Municipality	Direct wet mount, formol‐ether concentration and modified Ziehl–Neelsen stain	Humans (children < 5 years)	Helminths and protozoa	14%	Kpene et al. [49]
Kassena‐Nankana East Municipal	Kato–Katz	Humans (school children 5–17 years)	Helminth	25.20%	Dassah et al. [50]
Banda District	Formol‐ether concentration	Humans (school children 5–16 years)	Helminth	40.40%	Donkoh et al. [51]
Kumasi	Direct wet mount	Humans (children)	Helminth	2.03%	Ronald et al. [52]
Accra	Real‐time PCR	Humans (children ≤ 5 years)	Protozoa	—	Opintan et al. [53]
Asante Akim North Municipality	Direct wet mount	Humans (children)	Helminths and protozoa	10.90%	Nkrumah and Nguah [17]
Asante Akim North Municipality	Real‐time PCR	Humans (children ≤ 14 years)	Protozoa	5.20%	Eibach et al. [54]
Accra	Direct wet mount and formol‐ether concentration	Humans (pregnant women)	Helminths and protozoa	13%	Ayeh‐Kumi et al. [55, 56]
Kumasi	Kato–Katz and Baermann technique	Humans (pregnant women)	Helminth	9.10%	Yatich et al. [57]
Kumasi	Kato–Katz and Baermann technique	Humans (pregnant women)	Helminth	25.70%	Yatich et al. [58]
Kumasi	Direct wet mount	Humans (pregnant and nonpregnant women)	Helminth	5.60%	Agboli et al. [59]
Dangme East District	Direct wet mount and formol‐ether concentration	Humans (pregnant women)	Helminth	28.53%	Tay et al. [60]
Bolgatanga	Direct wet mount and formol‐ether concentration	Humans (pregnant women)	Helminths and protozoa	—	Ahenkorah et al. [61]
Kasoa	Direct wet mount and formol‐ether concentration	Humans (pregnant women)	Helminths and protozoa	14.30%	Abaka‐Yawson et al. [62]
Kumasi	Direct wet mount, formol‐ether concentration and staining	Humans (pregnant women)	Protozoa	36.70%	Atakorah et al. [63]
Accra	Direct wet mount and formol‐ether concentration	Humans (mentally challenged)	Helminths and protozoa	13.5%	Duedu et al. [64]
Accra	Formol‐ether concentration and Ziehl–Neelsen technique	Humans (immunocompromised patients)	Helminths and protozoa	35.42%	Adjei et al. [65]
Accra	Direct wet mount, formol‐ether concentration and modified Ziehl–Neelsen stain	Humans (immunocompromised patients)	Helminths and protozoa	23.50%	Boaitey et al. [66]
Cape Coast	ELISA	Humans (immunocompromised patients)	Protozoa	6.20%	Dankwa et al. [67]
Coastal savannah	Formol‐ether concentration and PCR	Humans (farmers)	Helminth	10.50%	Squire et al. [68]
Accra	Direct wet mount, formol‐ether concentration, trichrome and the modified Ziehl–Neelsen staining	Humans (food vendors)	Helminths and protozoa	21.60%	Ayeh‐Kumi et al. [55, 56]
Kumasi	Direct wet mount and formol‐ether concentration	Humans (food vendors)	Helminths and protozoa	78.60%	Adams and Lawson [69]
Tamale	Direct wet mount and formol‐ether concentration	Humans (food vendors)	Helminths and protozoa	34.70%	Danikuu et al. [70]
Pong‐Tamale and Ejura	Modified McMaster technique	Livestock (lamb)	Helminths and protozoa	—	Agyei et al. [71]
Pokuase	Modified McMaster technique	Livestock (cattle)	Helminth	—	Agyei [72]
Pokuase and Frafraha	Modified Ziehl–Neelsen acid‐fast staining technique	Livestock (cattle)	Protozoa	29.00%	Squire et al. [73]
Coastal savannah	Formol‐ether concentration	Livestock (cattle, sheep and goats)	Helminth	90.8%	Squire et al. [74]
Coastal savannah	Formol‐ether concentration and PCR	Livestock (cattle, sheep and goats)	Helminth	64.30%	Squire et al. [68]
Accra	Kato–Katz	Livestock (goat)	Helminth	100%	Futagbi et al. [75]
Kpong	Direct wet mount	Livestock (cattle)	Helminth	61.67%	Mensah et al. [76]
Kpong	Modified Ziehl–Neelsen technique and PCR	Livestock (cattle) and water samples	Protozoa	10%	Mensah et al. [77]
Upper East Region	McMaster technique, formol‐ether concentration	Livestock (pigs)	Helminths and protozoa	93.40%	Permin et al. [78]
Ga West Municipality	Direct wet mount	Humans (pig farmers and their household)	Protozoa	10.4%	Aninagyei et al. [79]
Ejisu‐Juaben Municipality	Formol‐ether concentration and modified Ziehl–Neelsen technique	Livestock (pigs)	Helminths and protozoa	76% for *Ascaris* and 77% for *Cryptosporidium*	Larbi et al. [80]
Upper Eastern Region	Direct wet mount	Livestock (chicken)	Helminth	100%	Poulsen et al. [81]
Accra	Formol‐ether concentration	Livestock (chicken)	Helminths and protozoa	79.60%	Ayeh‐Kumi et al. [19]
Kumasi	Flotation technique	Livestock (chicken)	Helminth	65.50%	Asumang et al. [82]
Mampong	Flotation technique	Dogs	Helminth	52.60%	Amissah‐Reynolds et al. [83]
Accra	—	Dogs	Helminth	—	Anteson and Corkish [84]
Accra	Modified McMaster technique	Dogs	Helminth	62.60%	[85]
Mole National Park	Direct wet mount and formol‐ether concentration	Baboons and warthogs	Helminths and protozoa	94.74% in baboons and 95.65% in warthogs	Larbi et al. [86]
Shai Hill Reserve	Direct wet mount and formol‐ether concentration	Baboons	Helminths and protozoa	92%	Larbi et al. [87]
Mole National Park	Faecal sedimentation	Baboons	Helminths and protozoa	93%	Ryan et al. [88]
Kumasi	Formol‐ether concentration	Farmers, soil and irrigation water	Helminth	—	Amoah et al. [89]
Accra	Direct wet mount, formol‐ether concentration, trichrome and the modified Ziehl–Neelsen staining	*Cyperus esculentus* L. (tiger nuts)	Helminths and protozoa	—	Ayeh‐Kumi et al. [90]
Koforidua	Direct wet mount and staining	Vegetables	Helminths and protozoa	57.50%	Kudah et al. [91]
Accra	Direct wet mount, Ziehl–Neelsen staining	Vegetables	Helminths and protozoa	—	Duedu et al. [92]
Accra	Direct wet mount	Ready‐to‐eat vegetable salads	Helminths and protozoa	32%	Amissah‐Reynolds et al. [93]
Ga East District	Concentration method	Soil, lettuce, irrigation water	Helminth	—	Mahami et al. [94]

### 2.1. Prevalence of Parasitic Infections in Children

Infections in children cause reduced absorption of nutrients leading to poor childhood development [95]. Studies suggest that children in day‐care centres are more susceptible to acquiring intestinal parasites, normally attributed to their unsanitary behaviours and indiscriminate ‘mouthing’ of items during playtime [96–98]. Statistical analysis indicates that treating children significantly reduces the burden of infections in the general population to 70% [99]. In Ghana, most studies have reported intestinal parasites (helminths and protozoa) to be prevalent in children from 0 to 17 years (Table 1). Some of the parasites causing infections include *G. duodenalis* [15, 17, 20, 45, 47], *Cryptosporidium parvum* and *Cryptosporidium hominis* [54], *S. mansoni* [50], *A. lumbricoides*, hookworm [16, 46] and *Dicrocoelium dendriticum* in children [52].

### 2.2. Intestinal Parasitic Infections in Pregnant Women

Intestinal parasites have been documented to have significant effects on pregnant women as a result of reduced immunity [100]. These parasites cause maternal anaemia and premature birth [101, 102], reduced pregnancy and birth weight and deficient foetal growth [103, 104]. Infections that occur during the first trimester are more severe than those occurring at later stages in pregnancy. In addition, women experience more severe infections during their first pregnancy as compared to subsequent ones [105]. Pregnant women in Ghana are often infected with helminths, *G. duodenalis* [55, 61] or *A. lumbricoides* [60]. Studies have also reported protozoa parasites such as *E. histolytica*/*dispar* and *C. parvum* to infect pregnant women [62, 63].

### 2.3. Prevalence of Intestinal Parasites in Other Vulnerable Groups

Amongst the vulnerable groups at risk of intestinal parasite infections are prison inmates. In developing countries, including Ghana, prisoners lack adequate facilities and resources, even access to potable water, leading to overcrowding and malnutrition [106]. They also have no control of their environment, hence a higher risk of infections caused by intestinal parasites [107]. It is safe to assume that infections could easily circulate amongst prisoners due to the prevailing conditions around them. To the best of our knowledge, only one study from Ghana has reported a high occurrence of intestinal parasites in prison inmates, prompting a public health concern [42]. Another institution that can facilitate the occurrence and spread of intestinal parasites is the psychiatric hospital. Overpopulation and reduced sanitary conditions in the hospital create a conducive environment for intestinal parasites to thrive and cause infections [108, 109]. A similar study was conducted in Ghana which revealed the risk of parasite transmission and a need for constant monitoring and intervention [64]. Infections caused by gastrointestinal parasites rank amongst the top causes of death for those with impaired immune systems, particularly those suffering from HIV [110]. In Ghana, there are reports of *Cryptosporidium* spp., *Strongyloides stercoralis*, *G. duodenalis* and hookworm infections in HIV patients [65–67].

## 3. Animal Parasitic Infections and Risk of Zoonotic Transmission

### 3.1. Livestock

**Figure 1:**
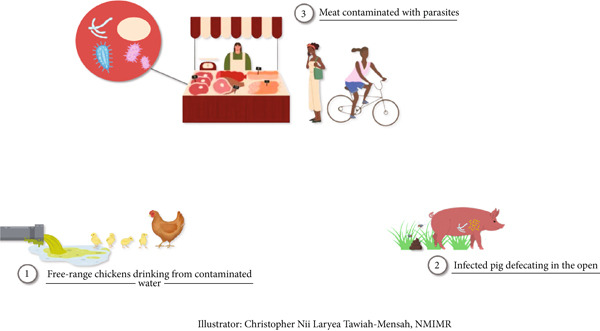
(1) The risk of parasitic infections such as *Cryptosporidium* spp. and *A. galli* due to exposure to infected water sources. (2) The transmission risk of zoonotic parasites like *T. solium* and *B. coli* from pig faeces contaminating the environment. (3) The public health risk of consuming improperly handled or undercooked meat infected with parasites such as *T. solium* or *Cryptosporidium* spp.

Livestock rearing has become a common practice in many Ghanaian communities. These animals are often kept either as a source of nutrition or for generating income and seldom as pets. There is however the risk of infections associated with keeping livestock [111]. In the tropics and subtropics, nematodes are responsible for most parasitic diseases in ruminant livestock [112]. Generally, infections with intestinal parasites have been associated with a decrease in animal production, leading to economic loss and appreciable mortality and morbidity [113]. It is thus important to prevent infections in the animals to boost production and further prevent the potential spread to farmers and individuals in proximity to the animals. In Ghana, there have been studies that address intestinal parasitic infections in ruminants [71, 74, 114–116]. Some studies have further suggested ways to control and reduce the burden of infections to increase ruminant production [117]. However, intestinal parasites continue to persist in ruminants within the country, increasing the risk of zoonotic transmission. An earlier study in Ghana reported the occurrence of nematodes including *Trichostrongylus axei* and *Trichostrongylus colubriformis* in sheep throughout the year [72]. These abovementioned nematodes have been reported to cause infections in humans [118]. A study in Southern Ghana reported a high occurrence of *Cryptosporidium* oocysts in cattle with an increased risk of zoonotic transmission to humans [73]. Additionally, goats slaughtered in an abattoir within Accra were highly infected with intestinal parasites, some of which could infect the workers [75]. In a more recent study, molecular characterization of *Trichostrongylus* species led investigators to suggest the zoonotic transmission of the parasites from infected cattle, sheep and goats [68]. Cattle from farms in Kpong were also identified to be infected with zoonotic intestinal parasites, thus increasing the risk of transmission to farmers and neighbouring inhabitants [76]. A follow‐up study resulted in the molecular identification of *C. parvum* in the sampled cattle [77]. The transmission of zoonotic intestinal parasites in Ghana needs critical attention to reduce the burden and public health risk (Figure 1).

Pigs are raised across Ghana for both commercial and nutritional reasons. However, pigs are a source of intestinal parasites, posing a risk to humans in close contact [119]. There have been reports of pigs infected with diverse intestinal parasites including *Balantidium coli* and *Taenia solium* which are zoonotic [78]. A follow‐up study found pigs and humans exposed to *Taenia* [120]. Other studies have reported *B. coli* in pig farmers [79] as well as *Ascaris* spp. and *Cryptosporidium* spp. in pigs, suggesting a potential transmission to the pig farmers [80]. Pig farmers and those in close contact are indeed at risk of infections; hence, they need to better protect themselves.

Evidence suggests that chicken production is significantly affected by gastrointestinal parasites. Due to the feeding habits of chickens, they may be exposed to infections when the food substance, water source and the environment harbour the infective stages of the parasites [121]. The situation in Ghana seems to be the same, as significant infections have been detected in faecal samples from sampled chickens. The parasites identified in chickens include *Raillietina echinobothrida*, *Raillietina tetragona*, *Gongylonema ingluvicola*, *Capillaria* spp., *Heterakis gallinarum* and *Ascaridia galli* [19, 81, 82].

### 3.2. Companion Animals

Although dogs as domestic pets provide companionship and security, they are known to transmit zoonotic pathogens such as *Toxocara canis* and *Ancylostoma* spp. [122]. In developing countries, dogs are commonly affected by gastrointestinal parasites [123] and are most likely to be transmitted to humans due to the lack of treatments and poor enforcement of pet ownership policies [124]. There is a paucity of data on intestinal parasites affecting dogs in Ghana. The first study carried out in Accra found helminth parasites in sampled dogs [84]. There were no other studies until 2015 when investigators obtained faecal samples from dogs in the Greater Accra Region and found the prevalence of gastrointestinal helminths to be 62.6% with the three zoonotic helminths being hookworms, *Toxocara* and *Dipylidium* [85]. Furthermore, a similar study conducted in the Ashanti Region determined the prevalence rate of helminth infections to be 52.6% in dogs [83]. There is a lack of knowledge on zoonosis and inappropriate pet care techniques, such as feeding dogs on the naked ground and inconsistent deworming. There are no published data on the prevalence of intestinal parasites in cats within Ghana. Since cats can also harbour and transmit *Toxocara* [122], it is essential to research and determine their role in the spread of intestinal parasites within the country and the risk of human infections.

### 3.3. Nonhuman Primates

Nonhuman primates are infected with intestinal parasites which can be transmitted to humans within a shared habitat [125–127]. Within a suitable environment and under favourable conditions, these zoonotic parasites significantly affect control efforts within the human population. In Ghana, there is a continuous interaction between humans and nonhuman primates, especially in and around tourist sites. A study within the Mole National Park in the Northern Region of Ghana revealed the potential zoonotic transmission of intestinal parasites from present baboons to inhabitants. The sampled baboons were found to be highly infected with intestinal parasites posing a health threat [88]. In a more recent study at the same park, sampled baboons and warthogs were found to be infected with multiple parasites with an increased risk of zoonotic transmission to nearby residents [86]. Furthermore, in the Shai Hills Reserve located within the Greater Accra Region of Ghana, baboons were infected with zoonotic intestinal parasites such as *A. lumbricoides*, *Diphyllobothrium latum* and hookworm [87]. Nonhuman primates within the country could pose a risk to public health and, as such, need to be monitored to prevent the spread of infections.

## 4. Intestinal Parasites in Fruits, Vegetables and Food Vendors

Even though fruits and vegetables are of nutritional value to consumers [128], they can serve as a source of intestinal parasitic infections [129, 130]. Contamination of fruits and vegetables can occur at any point from planting to consumption [131]. It is common for farmers in Ghana to use wastewater as a source of irrigation for crop production. When farmers rely on the use of wastewater irrigation, there is an increased risk of parasitic contamination and infection. This situation has been observed in Ghana, where farmers are exposed to soil‐transmitted helminths due to the use of wastewater for irrigation [89]. Thus, in Ghana, the raw consumption of fruits and vegetables significantly increases the risk of parasitic infections. In Accra, a study reported a risk to public health after identifying *C. parvum*, *S. stercoralis*, *Ancylostoma duodenale* and *C. cayetanensis* on *Cyperus esculentus* L. that are usually consumed raw [90]. Other studies have reported the occurrence of intestinal parasites on commonly consumed fruits and vegetables [91, 92], ready‐to‐eat salads [93] and lettuce produced using irrigated wastewater [94]. The findings suggest an increased risk to public health and the need to educate farmers and consumers.

Over the years, there has been an increasing number of people patronizing food from various food vendors. Worldwide, it is estimated that about 2.1 billion people frequent food vendors [132]. As such, under favourable conditions, food vendors and handlers can serve as a source of intestinal parasite transmission [133]. Studies in Ghana indicate food vendors are infected with both protozoa and helminths that can be transmitted to other humans under favourable conditions [56, 69, 70]. Thus, food vendors play a key role in intestinal parasite transmission within Ghana.

## 5. Control

In the absence of effective vaccines, preventive chemotherapy has been adopted as the approach to treat affected individuals and minimize transmission [97, 134]. However, the use of chemotherapy in treating intestinal parasites is insufficient to reduce the spread and burden of resulting diseases. The WHO has recommended some basic strategies to reduce the burden of intestinal parasites. These include education, the use of protective clothing in areas of high risk, the safe handling and disposal of human waste and improving the quality of hygiene, sanitation and water supply [11]. For the treatment of infections caused by intestinal protozoan parasites, some recommended drugs include metronidazole, paromomycin and diloxanide furoate [135]. Ghana as a country has employed the use of WHO‐recommended drugs for nationwide deworming exercises and periodically embarks on mass education targeting mostly basic schools [136]. To control soil‐transmitted helminths, especially in school children, WHO recommends the mass drug administration (MDA) of anthelmintics such as benzimidazoles (albendazole or mebendazole), annually or twice yearly [137]. Even though the MDA has been effective to an extent, there remain some setbacks. A typical example is MDA programmes that target children enrolled in formal education; only those with regular attendance will benefit to the detriment of irregularly attending children [138, 139]. Another issue has to do with drug resistance which has yet to be given major attention globally [140, 141]. In Ghana, benzimidazole resistance has been detected in human hookworms within the Central Region [142] which could help to explain why patients in Ghana seem to respond differently to benzimidazole‐based treatment [35, 143]. This highlights the need to conduct nationwide surveillance to establish the trend of drug resistance, especially in vulnerable populations, to create effective treatment strategies. Furthermore, as part of control and prevention efforts, improving the nutritional status of vulnerable populations will reduce the risk of infections [143].

Although most infections are noted to occur rather in rural areas, there seems to be an alarming paradigm shift in this trend. In that, owing to the recent rise in urban migration, overcrowding and the formation of slums and squatter settlements have become inevitable, with an increased incidence of polyparasitism of protozoa and helminths. Knowledge of this and further urban ecological studies would be essential control strategies to consider [97].

## 6. Discussion

The information provided in this review highlights the persistent occurrence of intestinal parasites, especially in children and people residing in rural areas with poor access to clean water and sanitation [144]. In settings with limited resources, these parasites continue to be a significant public health concern, which is consistent with global trends [145].

In Ghana, a major problem is the infections caused by both intestinal protozoa and helminths. Poor sanitation and hygiene standards are directly linked to these parasites, which are frequently spread by faecal–oral pathways [146]. These protozoan infections are highly prevalent, particularly in children, and can exacerbate already existing health disparities by causing persistent diarrhoea, malnutrition and impaired cognitive development [147, 148]. Pregnant women also face the risk of anaemia when infected with intestinal parasites [60]. With regular screening, monitoring and the administration of effective drugs to infected individuals, there would be an improvement in the health of both expectant mothers and their babies.

The emphasis on zoonotic intestinal parasite infections is a crucial component of this review. In Ghana, parasites such as hookworm and *C. parvum* are found in both human and animal populations, demonstrating the connection between animal and human health [44, 77]. Furthermore, baboons present in tourist sites such as the Mole National Park and Shai Hill Reserve harbour intestinal parasites that can infect humans [86, 87]. A favourable environment for zoonotic transmission is produced by the proximity of wild and domestic animals to human homes, as well as by poor sanitation and hygiene standards [149]. Where animal production is the main source of income, the potential of zoonotic parasitic infections is especially worrisome. These parasites are disseminated through handling animal excrement and eating raw or undercooked meat [150]. Additionally, wandering dogs and cats raise the possibility of zoonotic parasite contamination of the environment in both urban and rural regions. When dealing with zoonotic intestinal parasites in Ghana, the One Health strategy is essential. This method highlights the necessity of cooperation in the fields of environmental, animal and human health. The risk of zoonotic transmission can be successfully decreased by putting integrated interventions into practice, such as cooperative research, coordinated surveillance and joint health education campaigns [151].

Ghana faces challenges in controlling intestinal parasite infections. The persistence of these infections is exacerbated by socioeconomic inequality, poor health education and restricted access to sanitary facilities and clean water. Furthermore, prompt identification and management of intestinal parasites are hampered by the absence of reliable surveillance systems and diagnostic capabilities [152]. Another obstacle to successful treatment is the development of drug resistance in certain parasites, especially protozoa [153]. Resistance may arise as a result of the careless use of antiparasitic medications and noncompliance with treatment recommendations. By addressing these gaps, Ghana can reduce the burden of intestinal parasites and improve the health of its inhabitants.

## 7. Conclusion

Infections caused by intestinal parasites are common in the poor populations of endemic regions. The ultimate goal is to reduce the effects of these infections to minimum levels to no longer be of public health importance. In achieving this, it is important to administer recommended drugs such as albendazole through healthcare facilities, embark on educational health campaigns, provide education on environmental management strategies and directly target and reach out to vulnerable groups within communities [154]. It is also important to conduct region‐wide education on improving sanitation, as this will reduce the spread of infections. The government cannot be left out in the quest to eliminate prevalence, as it has a role to play in ensuring good housing as well as the supply of treated potable drinking water to homes, especially in rural communities where the burden is on the rise.

## 8. Future

It is recommended that future studies should focus on the vulnerable populations within the country to establish the true burden of infections as this will aid in the formulation of effective control strategies. Deworming exercises should also be included in the routine activities of health facilities if not already in place and the education of patients, as well as all visitors, be intensified. Carrying out effective monitoring and evaluation of the MDA is pivotal in ascertaining the possibility of emerging drug resistance and incidence of posttreatment reinfection. There is a need to conduct more studies on the role of animals in the transmission of intestinal parasites. Reducing the burden of infections in the animal host and subsequent introduction of regulations regarding the possession of animals will ultimately bring zoonosis to the barest minimum.

## Conflicts of Interest

The authors declare no conflicts of interest.

## Funding

No funding was received for this manuscript.

## Data Availability

Data sharing is not applicable to this article as no datasets were generated or analyzed during the current study.
